# The incidence of type 1 diabetes mellitus among 15-34 years aged Lithuanian population: 18-year incidence study based on prospective databases

**DOI:** 10.1186/1471-2458-11-813

**Published:** 2011-10-19

**Authors:** Rytas Ostrauskas, Rimantas Žalinkevičius, Nijolė Jurgevičienė, Lina Radzevičienė, Lina Lašaitė

**Affiliations:** 1 Institute of Endocrinology, Lithuanian University of Health Sciences, Eiveniu 2, Kaunas, LT 50009, Lithuania

## Abstract

**Background:**

The aim of this prospective study was to determine the incidence of type 1 diabetes mellitus in 15-34-year-aged Lithuanian males and females during 1991 - 2008

**Methods:**

A contact system with general practitioners covering 100% of the 15-34-year-aged Lithuanian population was the primary data source. Reports from regional endocrinologists and statistical note-marks of State patient insurance fund served as secondary sources for case ascertainment.

**Results:**

The average age-standardized incidence rate was 8.30 per 100,000 persons per year (95% Poisson distribution confidence interval [CI] 7.90-8.71) during 1991 - 2008 and was statistically significantly higher among males (10.44 per 100,000 persons per year, 95% CI 9.82-11.10) in comparison with females (6.10 per 100,000, 95% CI 5.62-6.62). Male/female rate ratio was 1.71 (95% CI 1.63-1.80). Results of the linear 1991 - 2008 regression model showed that the incidence of Type 1 diabetes in 15-34-year-aged males and females decreased slightly over the time (r = -0.215, p > 0.05).

**Conclusions:**

Our data demonstrated the male predominance in primary incidence of type 1 diabetes mellitus in 15-34-year-aged population in Lithuania. The incidence of type 1 diabetes mellitus in 15-34-year-aged males and females decreased slightly during 1991-2008.

## Background

Type 1 diabetes mellitus is caused by non-genetic, probably environmental, factors operating in a genetically susceptible host to initiate a destructive immune process [1]. Despite of the well-established role of HLA genetics in the aetiology of type 1 diabetes [2,3] and several environmental factors suggested to be related to beta-cell destruction [4], the aetiology of type 1 diabetes is still unclear. In children type 1 diabetes usually have been considered as multifactorial disease in which environmental risk factors trigger an immune-mediated destruction of the pancreatic beta cells in genetically susceptible persons [5]. The critical period of immune activation is probably short and the process leading to diabetes has a long prodrome but of variable duration that determines the age at presentation with clinical disease. Type 1 diabetes presenting in adults, in contrast to children, have been predominantly determined by non-genetic factors with a reduced capacity for protective and susceptible HLA alleles [5]. In childhood type 1 diabetes presents with acute, life-threatening, symptomatic hyperglycaemia. In 15-year and older adolescents and adults the symptoms of diabetes are typically less acute and the period from the beginning of symptoms to development of the disease is longer [6]. However, the onset of type 1 diabetes in adulthood requires no less attention than in childhood.

In most countries, 0-15-year-aged children with diabetes present at paediatric services and are highly visible with the heath care system [7]. The highest reported incidence rates are in Finland, with increasing from 31.4 per 100,000 inhabitants per year in 1980 to 64.2 per 100,000 inhabitants per year in 2005, and the lowest (0.1 per 100,000 inhabitants per year) among children under the age of 15 years in China and Venezuela, the states far one to another [7,8]. Large variations in the incidence rates of 0-14-year-aged type 1 diabetes are also seen in the countries of the Baltic Sea region [9]. There are limited worldwide data allowing evaluating geographical and temporal trends for the risk of developing type 1 diabetes in adults [8,10-15], and only one international comparison of type 1 diabetes incidence among 15-29-year-aged inhabitants from 9 study centres of Europe [6]. A prospective register of newly diagnosed cases of type 1 diabetes in 15-year and older adults was established in Lithuania in 1991. Lithuania is a state on the eastern coast of the Baltic Sea. Area - 65,300 (sq) km. Neighbour states are Latvia, Belarus, Poland, and Russia. The total population at the end of 2008 was 3,349,872 (1,559,247 males and 1,790,625 females). The proportion of native Lithuanians was 83.5%. Other ethnic groups were Poles (6.7%), Russians (6.3%), and 3.5% - one hundred eleven other ethnicities [16].

Herein we report incidence data got prospectively during the period from 1991 to 2008.

## Methods

### Data Source

Since 1 January 1991, patients with type 1 diabetes between 15 and 34 years of age and permanently residing in Lithuania have been prospectively registered. All physicians responsible for outpatient care of people with diabetes report new cases with type 1 diabetes to the data-collecting centre in the special form that is deemed mandatory by the Lithuanian Ministry of Health. Information including personal identification code, name, date of birth, sex, address, date of clinical diagnosis, date of first insulin injection, reporting unit and physician, and some clinical characteristics (ketonuria and/or acidosis, blood glucose value at the time of diagnosis), was registered for every patient. Diabetes was defined according to the World Health Organization Criteria [17]. Only those cases where insulin therapy was initiated at or within 2 weeks of diagnosis, and lasted for at least several months, were regarded as having type 1 diabetes and included into the registry. The date of the first insulin injection was used as date of diagnoses in the analyses. New cases reported throughout the previous year were checked entering into the lists of patients to verify that they still are treated with insulin and to find the cases that were missed. These lists are considered as the secondary source of ascertainment together with records of the insurance statistical note-marks of diabetic patients who visited Medical Units.

The criteria for diagnosing type 1 diabetes in children were generally recognized for diabetes registries, which also are those of the DiaMond protocol [18]: diagnosis of idiopathic diabetes stated by a physician, with insulin therapy started before the 15th birthday in the believe that long-term treatment with insulin will be necessary. In international study performed in Europe, the criteria for the diagnosis of type 1 diabetes among the young 15-29-year-aged adult population were the same, apart from the age limit of insulin therapy, obviously, years of age [6].

According to the current practice in Lithuania, cases characterized by low or normal body weight, young age, severe hyperglycaemia, ketosis and an immediate need for insulin therapy are regarded as type 1. Obesity, few symptoms and a considerable improvement following dietary measures, alone or in combination with oral treatment, indicate the presence of type 2 diabetes mellitus. In the absence of a universally accepted epidemiological definition of type 1 diabetes in 15-34-year-aged young adult patients additional criteria for eligibility for the present study were used: age at onset <34 years, and insulin treatment known to have commenced at diagnosis. One of the main conditions for inclusion into the register was: type 1 diabetes was defined as the presence of ketonuria, and the need for permanent insulin therapy [6]. Patients with secondary diabetes, type 2 diabetes, gestational diabetes, and chemotherapy-induced diabetes were excluded. In the present study, in which the classification was based more on the clinical experience of the reporting physician, a certain degree of misclassification may be expected. More detailed registration procedure of type 1 diabetes in adulthood in Lithuania has been published previously [19].

### Statistical analyses

Information from all sources was entered into the computer based information system. Capture-recapture method was employed to detect the number of missing cases and to adjust count for accurately estimated number of people who had diabetes [20].

The Statistica 8.0 and SPSS+14.0 software packages were used for statistical analysis of the data. Incidence rates were calculated using denominated data of Lithuanian Statistical Department [16]. The incidence rates were calculated per 100,000 persons per year. The average incidence rates per year, for an 18-year period 1991-2008, were calculated for males and females separately and together in the age groups 15-19, 20-24, 25-29, and 30-34 years. Direct age standardisation of the incidence rates was performed assuming a standard population with equally sizes 5-year age groups of both genders. For comparison with data from other areas the incidence rates, standardization was performed by using World Standard Population [21]. For determining differences between the gender and age groups, χ^2 ^was used [22,23]. The limit of significance was defined as a p-value lower than 0.05. The 95% confidence intervals (CI) were estimated assuming Poisson distribution of the cases [22,24]. Time-series analysis was used to evaluate the changing pattern of the incidence of type 1 diabetes [23].

The study was approved by Kaunas Regional Ethics Committee of Biomedical Researches (No. BE-2-63).

## Results

The number of incident 15-34-year-aged type 1 diabetes cases diagnosed in Lithuania from 1 January 1991 to 31 December 2008 was 1591 (1014 males and 577 females) (Table 1). The overall completeness of case ascertainment was estimated at 86.83% in 15-34-year-aged Lithuanian inhabitants. Throughout the secondary sources of case-ascertainment 182 additional cases of type 1 diabetes were identified during the 18-year period.

**Table 1:** Age-specific average Type 1 diabetes mellitus incidence rates (per 100,000 persons per year) in Lithuania between 1991 and 2008.

	Males				Females				Both			
	
			Incidence				Incidence				Incidence	
									
Age group (years)	Cases	Mean population per year	Rate	95% CI	Cases	Mean population per year	Rate	95% CI	Cases	Mean population per year	Rate	95% CI
15-19	183	135,942	7.48	6.47-8.65	175	131,486	7.39	6.37-8.57	358	267,427	7.44	6.71-8.25
20-24	212	135,439	8.70	7.60-9.95	129	130,685	5.48	4.61-6.51	341	266,107	7.12	6.40-7.92
25-29	284	133,085	11.86	10.56-13.32	130	128,366	5.63	4.74-6.69	414	261,451	8.88	8.06-9.78
30-34	335	135,605	13.72	12.33-15.27	143	135,204	5.88	4.99-6.93	478	270,809	9.81	8.97-10.73

Total	1014	540,071	10.43	8.91-11.09	577	525,740	6.17	5.69-6.69	1591	1,065,795	8.13	7.74-8.54

15-24*	395	271,381	8.05	7.29-8.88	304	262,170	6.49	5.80-7.26	699	533,535	7.29	6.77-7.85
15-29*	679	404,466	9.27	8.60-9.99	434	390,536	6.22	5.66-6.83	1113	794,986	7.80	7.35-8.27
15-34*	1014	540,071	10.13	9.53-10.77	577	525,740	6.15	5.66-6.67	1591	1,065,795	8.19	7.80-8.60

*Incidence rates age-adjustment using World Standard Population

The average age-standardized 18-year incidence rates in entire 15-34-year age group was 8.30 per 100,000 persons per year (95% Poisson distribution confidence interval [CI] 7.90-8.71), and a trend was found towards pointedly higher incidence among males (10.44 per 100,000 persons per year, 95% CI 9.82-11.10) in comparison with females (6.10 per 100,000 persons per year, 95% CI 5.62-6.62). χ^2 ^gender difference was 493.99, df = 3, p < 0.000001.

**Figure 1:**
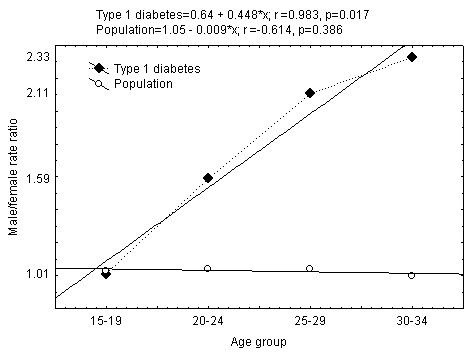
**Average male/female incidence rate per 100.000 per year ratio variation and trend in different age groups between 1991 and 2008**.

Figure 1 presents the average male/female incidence rates ratio in 5-year age groups. Male/female ratio in entire 15-34-year-age group was 1.71 (95% CI 1.63-1.80). Ratio of total male/female population was 1.03.

**Figure 2:**
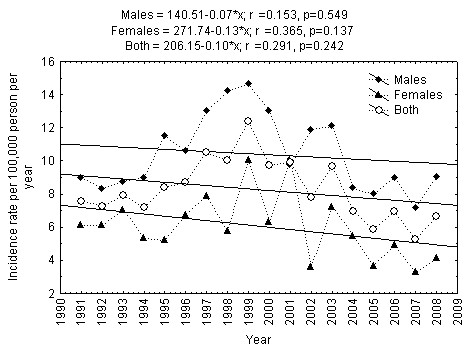
**Trend in incidence of Type 1 diabetes mellitus in 15-34-year-aged Lithuanian inhabitants during 1991-2008**.

The incidence rates and regression based linear trends of type 1 diabetes in 15-34-year-aged males, females, and all Lithuania inhabitants during 1991-2008 are presented in Figure 2. Results of the regression models showed that the incidence of type 1 diabetes in 15-34-year-aged Lithuanian males and females during all 1991-2008 year period decreased slightly over time. The highest incidence of type 1 diabetes in males and females was observed in 1999 (14.62 per 100,000 person per year among males and 10.06 per 100,000 person per year among females, p > 0.05), and the lowest in 2007 (8.03 and 2.78 per 100,000 person per year among males and females, respectively, p < 0.01).

**Figure 3:**
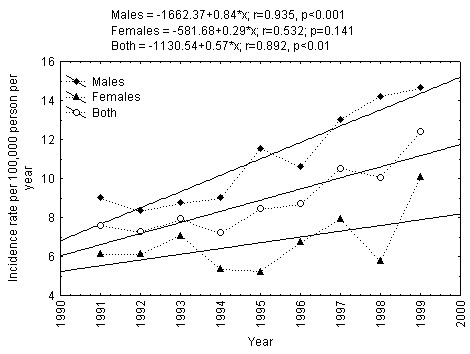
**Trend in incidence of Type 1 diabetes mellitus in 15-34-year-aged Lithuanian inhabitants during 1991-1999**.

**Figure 4:**
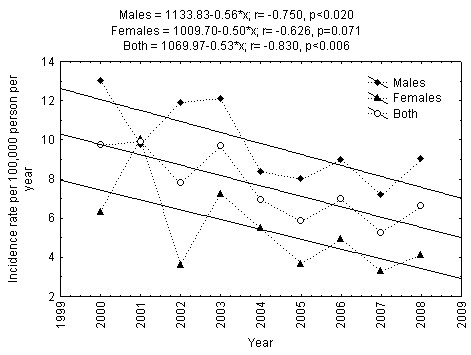
**Trend in incidence of Type 1 diabetes mellitus in 15-34-year-aged Lithuanian inhabitants during 2000-2008**.

The period of 1991-2008 may be divided into two equal nine-year periods: 1991-1999 and 2000-2008. The regression based linear trends showed that the incidence of type 1 diabetes among males increased statistically significantly among males (p < 0.001), but not among females during 1991-1999 in Lithuania (Figure 3). The incidence of type 1 diabetes among males decreased statistically significantly (p < 0.02), while the changes in incidence of type 1 diabetes among females remained relatively stable during 2000-2008 (Figure 4). However, in case of exclusion of the male type 1 diabetes incidence rate of 2000, the incidence trend among 15-34-year-aged males remained relatively stable during 2001-2008 (r = -0.632, p = 0.093).

## Discussion

Epidemiologically based standardized surveillance systems are needed for studying the time trends in the incidence of type 1 diabetes in childhood and adulthood, as they provide knowledge on the pattern of the disease occurrence and prevalence of the potential etiological factors.

The epidemiology of type 1 diabetes in adults is less well characterized than in 0-14-year-aged children, especially with regard to international comparison of incidence rates and to differences in incidence according to gender. The rising incidence of type 1 diabetes globally suggests the need for continuous monitoring of incidence by using standardized methods in order to plan or assess prevention strategies [7]. Epidemiological studies of type 1 diabetes mellitus in the Baltic Sea Basin population are important because of the large variation in childhood onset diabetes incidence recorded over the region [8,25-27]. The average of 24-year (1983-2007) type 1 diabetes incidence rates among 0-4, 5-9, 10-14, and all 0-14-year-aged Lithuanian children was 4.9, 9.1, 11.4, and 8.8 per 100,000 persons per year, respectively (5.3, 7.8, 12.2, 8.6 and 4.6, 10.3, 11.7, 8.4 per 100,000 persons per year among boys and girls, respectively). The proportion of new onset type 1 diabetic boys and girls was 0.95, p < 0.001. The incidence of type 1 diabetes increased with age and was the highest among 10-14-year-aged children: 11.9 (95% CI 8.2-9.1) per 100,000 person years. The average of annual incidence rate increase in 0-14-year-aged Lithuanian children was 3.6% during 1983-2007 in Lithuania [28].

In spite of the fact that the incidence rates of type 1 diabetes among 0-14-year-aged children are fairly well known worldwide [7,29], the incidence data of type 1 diabetes among young adults are poorly presented.

### Incidence of type 1 diabetes in adults - comparison with other countries

Up till now only one collaborative research project report about the studied incidence of type 1 diabetes in the 15-29-year-aged group in nine European regions known to represent high, intermediate and low childhood type 1 diabetes incidence areas was published [6]. Concerning this study the standardized incidence rate among 15-29-year aged Lithuanian inhabitants for 1996-1997 was lower than in Sardinia, Italy (p < 0.003), Catalonia, Spain (p < 0.05), Leicestershire, UK (p < 0.01), West Yorkshire, UK (p < 0.01), Sweden (p < 0.02), higher than in Slovakia (p < 0,002), and statistically similar as in Antwerp, Belgium, and Bucharest, Romania. This study showed that the incidence of type 1 diabetes among 15-29-year-aged young adults had less variation than among 0-14-year aged children from the same regions. In all nine centres the estimated male to female ratio of type 1 diabetes incidence in young adults was higher than one and higher than in 0-14-ear-aged children [6,7].

The highest incidence rates of type 1 diabetes in the World among Finish adult inhabitants were ascertained during 1992-1996. The incidence rates of type 1 diabetes among 15-19, 20-24, 25-29, and 30-34-year-aged Finish population was 22.5, 16.1, 16.2, and 15.2 per 100,000 person years, respectively (27.1, 19.9, 20.9, 19.8 and 17.6, 12.2, 11.3, 10.3 per 100,000 person years among males and females, respectively) [14]. In Upper Austria (1994-1996), Catalonia, Spain (1989-1998), Turin, Italy (1991-2001), Northern Italy (1984-2004) the average incidence rates of type 1 diabetes among 15-29-year-aged inhabitants were correspondingly 7.1 (95% CI 5.5-9.0), 10.2 (95%CI 9.7-10.8), 6.8 (95%CI 6.3-7.4) and 7.1 (95% CI 6.6-7.7) per 100.000 person years, and like in Lithuania higher than among 0-14-year-aged children [10,11,30,31]. Average annual increases in incidence rates were similar in children and young adults in Northern Italy, not supporting the hypothesis of a shift towards younger age as the main explanation for the increasing temporal trend in children [10]. In Turin, Italy it was observed that the risk of type 1 diabetes between age 30 and 49 years was similar to that found in the same area between age 15 and 29 years [11]. Evaluation of the incidence rate of the type 1 diabetes in 10 selected areas during 1998-2000 in Poland revealed values from 8.4 to 14.7 per 100,000 person years in the age group 1-14 and from 4.4 to 11.2 per 100,000 person years in the age group 15-29 [12]. It accounts for the 2-3-fold increase in comparison with the results achieved in 1986 [32].

Observed countries-related differences in type 1 diabetes incidence rates probably were correlated to host/genetic factors or environmental (virus/diet) agents in different geographical areas [33-36]. In Lithuanian case-control study of 124 children with diabetes participants were tested for HLA class II and compared with 78 healthy controls. Authors conclude that HLA class II haplotypes associated with type 1 diabetes positively or negatively were the same in Lithuanian children as in other European Caucasian populations, and differences in the incidence and clinical manifestations of type 1 diabetes might be due to different environmental factors and/or lifestyle [37].

### Gender differences in the incidence of type 1 diabetes in adults

The average incidence rates of type 1 diabetes in Lithuania were lower among females than among males in 20-24, 25-29, and 30-34-year-aged groups. A clear male predominance of type 1 diabetes was seen in all ages during 1983-2002 in Sweden. The average incidence rate of type 1 diabetes was 12.7 per 100,000 person years (16.4 and 8.9 among males and females) [15]. The male predominance in the majority of 24 populations and the female excess in 9 of them were well discussed by Östman et al. in 2007 [15]. The male excess was not related to the high, intermediate or low level of the incidence [15]. In Lithuania, the average incidence rates of 1983-2007 year of type 1 diabetes in 0-4-year-aged group among boys and girls did not differ significantly, in 5-9-year-aged group the incidence of type 1 diabetes was significantly higher among girls than among boys. The disappearance of gender difference starts during the puberty in 10-14-year-aged group [28]. The results of the present study showed that significant male predominance of the incidence rates of type 1 diabetes started in 20-24-year-aged group, and increased with the age of the onset of disease. The highest gender difference with male predominance was observed in 30-34-year-aged group in Lithuania. We did not find any possible cause of under-reported cases among females in 15-34-year-aged group. Environmental exposures may differ for females and males. The results of Swedish study suggest that gestational enterovirus infections may be related to the risk of the offspring developing type 1 diabetes in adolescence and young adulthood. Boys of enterovirus IgM-positive mothers had approximately 5 times greater risk of developing diabetes, as compared to boys of IgM-negative mothers [38]. There are innate differences in the function of the female and male immune systems, and there is some evidence for differences between females and males in the ability of a target organ for autoimmunity to withstand damage [39]. Besides a genetic basis, sex hormones affect the function of the mammalian immune system and make undeniable contributions to the sexually dimorphic expression of autoimmune disease [40]. Sex hormones seem to be secondary players that are responsible for helping in a process that may have been triggered by others. Androgen promotes autoimmune diseases with a profile of type 1 cytokines [41]. The poor induction of factors that mediate down-modulation of T-cell responses upon stimulation in type 1 cytokine environment may contribute to the development of autoreactive type 1 responses in the target tissue of type 1 diabetes [42]. Indeed, it suggests that males are more susceptible to environmental agents. Male excess has previously been reported and was largely restricted to those patients carrying the HLA-DR3/nonDR4 genotype [43]. Results of another study do not support a significant involvement of the Y chromosome in DR3/nonDR4 type 1 diabetic cases in early-onset type 1 diabetes as a whole [44]. Other explanations, such as X chromosome-linked inheritance, are thus required for the male bias in incidence in type 1 diabetes in Sardinia [44].

### Temporal trends in the incidence of type 1 diabetes in adults

The incidence of type 1 diabetes has increased between 1991 and 1999 in 15-34-year-aged Lithuanian inhabitants. Independently on gender, the incidence of type 1 diabetes has decreased during 2000-2008 years observation period. The incidence of type 1 diabetes was relatively stable, but with statistically insignificant tendency to decline during all (1991-2008) observation period in 15-34-year-aged young adults, but not in 0-14-year-aged children in Lithuania [28]. The declining incidence of type 1 diabetes was observed in 20-year prospective, nationwide study from Sweden among 15-34-year-aged young adults [15]. In Sweden, in the 15-34-year age group the incidence rate during the 20 years period decreased significantly in men, but not in women [15]. In Antwerp, Belgium type 1 diabetes in the age group of 0-39 years has not increased between 1989 and 2000 [14]. In Yorkshire (UK), a steady and continuing rise in the incidence of type 1 diabetes over time is observed for 0-14-year-aged children from 1978 to 2000, but not for young 15-29-year-aged adults during 1991-1999. A steady and continuing rise in the incidence of type 1 diabetes over time was observed for children but not for young adults [45].

Type 1 diabetes national/regional registries in 0-14-year-aged children represents an approximately 400-fold incidence in the over 100 populations/countries studied. In some countries having data from more than two registries, a marked within-country variation incidence has been reported [46]. For example, the variation in the incidence in four Italian regions was more than five-fold [7]. Some of the regions bordering Finland show up to 6-fold lower type 1 diabetes incidence rates than Finland itself.

Although Finland and Estonia are quite similar ethnically, the incidence of type 1 diabetes in Estonia is closer to the level seen in Lithuania and Latvia than in Finland. In order to explain the existing differences in the incidence by genetic means, HLA genotyping of multiplex diabetic families (with a type 1 diabetes child diagnosed less than 15 years of age) from Finland and Estonia has been performed. No Estonian diabetic patients possessed the A2, C1, B56, DR4, DQ8 haplotype, which is third most common, transmitted "diabetic" haplotype in Finland with highest absolute risk of type 1 diabetes. Most probably, Finns and Estonians have quite different genetic susceptibility for type 1 diabetes [47]. Therefore more by hypothetical than by objective reasons countries and study regions with well established registries are divided in very high, high, intermediate and low type 1 incident populations [7,48]. As almost all the countries of Central Europe, Lithuania with the incidence rate of type 1 diabetes among 0-14-year-aged children falls into the group of territories with intermediate incidence rates [7,25]. In lack of worldwide incidence data about type 1 diabetes in adulthood, we only can suppose, that incidence rates of type 1 diabetes in young adults comparing with other countries probably in Lithuania may be approximately the same. As in above mentioned studies from Sweden and Belgium [14,15], the stabilization of time trend of incidence rates in young adults in Lithuania with increasing incidence rates in Lithuanian children may be related with a shift to younger age at the onset of type 1 diabetes.

Huge emigration of the native population may be another reason for declining results of time trend of type 1 diabetes incidence in adulthood in Lithuania. After the restoration of Lithuania's independence, immigration flows began to decrease, while emigration - increased considerably. The decrease in the number of the Lithuanian population was determined by negative net migration. Net migration in 2001 2002, 2003, 2004, 2005, 2006, 2007 and 2008 was respectively -0.7, -0.6, -1.8, -2.8, -2.6, -1.4, -1.5 and -2.3 per 1000 Lithuanian inhabitants per year [49]. In 2008, the lowest negative net migration per 1000 population was recorded only in five EU Member States: Lithuania (-2.3), Latvia (-1.1), Germany (-0.7), Poland (-0.4), Latvia (-0.3), and Bulgaria (-0.1). The recent migration situation in Lithuania has been impacted by a deteriorating economic and financial situation, rapidly growing unemployment and a declining number of job vacancies. People lost due to emigration are mostly of the youth age 25-29. In 2008, a 20% of all Lithuanian emigrants were aged 25-29 years, 13% - 20-24 years, 14% - 30-34 years (in 2005, 21%, 16% and 13% respectively) [49]. The additional epidemiological studies are needed in order to obtain data on the incidence of type 1 diabetes among emigrants from Lithuania.

### Strengths and limitations of the study

The varying incidence rates observed in different countries were probably only partly due to methodological differences in the studies under comparison. Most of above-mentioned studies were characterized by a high level of ascertainment, which should contribute to a reduction of methodological differences. We were not able to find published data of similar registers, which cover the age range of 15-29 or 15-34 years during 2004-2008.

We think that the knowledge of the incidence rates of type 1 diabetes in Lithuania could contribute to the studies of a role of genetic and environmental factors that may be important in development of type 1 diabetes. The Lithuanian type 1 diabetes mellitus register may serve as the basis for prospective research.

## Conclusions

Our data demonstrated the male predominance in primary incidence of type 1 diabetes mellitus in 15-34-year-aged population in Lithuania. The incidence of type 1 diabetes mellitus in 15-34-year-aged males and females decreased slightly during 1991-2008.

## Competing interests

The authors declare that they have no competing interests.

## Authors' contributions

RO designed the study, researched data, contributed to discussion, wrote manuscript, edited manuscript. RZ researched data, contributed to discussion, wrote manuscript, edited manuscript. NJ researched data. LR researched data, contributed to discussion, reviewed/edited manuscript. LL researched data, reviewed/edited manuscript. All authors read and approved the final manuscript.

## Pre-publication history

The pre-publication history for this paper can be accessed here:

http://www.biomedcentral.com/1471-2458/11/813/prepub
